# Bayesian and Markov chain Monte Carlo methods for identifying nonlinear systems in the presence of uncertainty

**DOI:** 10.1098/rsta.2014.0405

**Published:** 2015-09-28

**Authors:** P. L. Green, K. Worden

**Affiliations:** Department of Mechanical Engineering, University of Sheffield, Mappin Street, Sheffield S1 3JD, UK

**Keywords:** nonlinear, system identification, model updating, Bayesian

## Abstract

In this paper, the authors outline the general principles behind an approach to Bayesian system identification and highlight the benefits of adopting a Bayesian framework when attempting to identify models of nonlinear dynamical systems in the presence of uncertainty. It is then described how, through a summary of some key algorithms, many of the potential difficulties associated with a Bayesian approach can be overcome through the use of Markov chain Monte Carlo (MCMC) methods. The paper concludes with a case study, where an MCMC algorithm is used to facilitate the Bayesian system identification of a nonlinear dynamical system from experimentally observed acceleration time histories.

## 1. Introduction

System Identification (SI) is a technique of considerable importance within the discipline of structural dynamics. In the absence of a complete physics-based description of a system or structure, SI can provide the missing pieces of information that allow the formulation of a descriptive or predictive model. When the structure of interest has linear dynamical behaviour, the problem of SI is well established, to the extent that authoritative text books and monographs exist [1,2]. In the case of linear dynamical systems, it is usually sufficient to consider sets of linear second-order differential equations (*modal* models) or first-order differential equations (*state-space* models) as the appropriate mathematical model structure. In that case, the SI problem is largely reduced to determining the correct number of equations and the numerical parameters in the model. Unfortunately, most structures will, in reality, display nonlinear characteristics to some extent, and the SI problem for nonlinear structures and systems is by no means solved. One of the main problems in nonlinear SI is the number and variety of possible model structures that arise once the variety of possible nonlinearities is taken into account [3,4].

It is not necessary here to provide a detailed classification of nonlinear SI models and approaches; however, it will prove useful to give a higher level breakdown of model structures based on their motivation. Predictive models can be divided into three classes: *white*, *grey* and *black-box* models.

*White-box* models are taken here to be those whose equations of motion have been derived completely from the underlying physics of the problem of interest and in which the model parameters have direct physical meanings. Finite-element models constitute one sub-class of such models.
*Black-box* models are, by contrast, usually formed by adopting a parametrized class of models with some universal approximation property and learning the parameters from measured data; in such a model, like a neural network, the parameters will not generally carry any physical meaning.
*Grey-box* models, as the name suggests, are usually a hybrid of the first two types above. They are commonly formed by taking a basic core motivated by known physics and then adding a black-box component with approximation properties suited to the problem of interest. A good example of a grey-box model is the Bouc–Wen model of hysteresis. In the Bouc–Wen model, a mass-spring-damper core is supplemented by an extra state-space equation which allows versatile approximation of a class of hysteresis loops [5,6].

In all of these cases, measured data from the system or structure of interest can be used in order to determine any unknown aspects of the model, e.g. any necessary undetermined parameters can be estimated. The use of measured data often means that uncertainty is introduced into the problem. There are two main sources of uncertainty caused by consideration of measured data. The first source is measurement noise; in general, other sources (*noise*) will contribute to measurements of the variable of interest and the direct distinction between signal and noise will be impossible. The second problem is encountered when a measured variable is itself a random process. In this case, only specific finite realizations of the process of interest can be measured; variability between realizations leads to variability between parameter estimates and thus gives rise to uncertainty.

In the past, the SI practitioner would generally implement the classical algorithms (i.e. least-squares minimization) as an exercise in linear algebra and would usually treat the resulting set of crisp parameter estimates as determining ‘the model’. Even if a covariance matrix were extracted, the user would usually use this only to provide confidence intervals or ‘error bars’ on the parameters; predictions would still be made using the crisp parameters produced by the algorithm. Such approaches do not fully accommodate the fact that a given set of measured data, subject to the sources of uncertainty discussed above, may be consistent with a number of different parametric models. It is now becoming clear—largely as a result of the pioneering work of James Beck and colleagues and more recently from guidance from the machine learning community—that a more robust approach to parameter estimation, and also model selection, can be formulated on the basis of Bayesian principles for probability and statistics. Among the potential advantages offered by a Bayesian formulation are the estimation procedure will return parameter distributions rather than parameters; predictions can be made by integrating over all parameters consistent with the data, weighted by their probabilities; evidence for a given model structure can be computed, leading to a principled means of model selection.

Adoption of Bayesian methods first became widespread in the context of the identification of black-box models; the methods have recently begun to occupy a central position within the machine learning community [7,8]. Bayesian methods for training multi-layer perceptron neural networks are a good example of this trend [9]; the Gaussian process model is also achieving wide popularity [10]. Most machine learning algorithms, like the neural networks and Gaussian processes already mentioned, are used to learn static relationships between variables; however, they can easily be used to model dynamical processes by assuming a NARX or NARMAX form for the mapping of interest [11,12]. A recent example of nonlinear system identification using Gaussian process NARX models can be found in [13]; this study is of interest because it shows how physical insight might be gained from the black-box GP NARX models. There has also been a body of work concerned with Bayesian parameter estimation for polynomial NARMAX models, a recent contribution can be found in [14].

In the context of white-box models, and in particular within the nonlinear SI community, the use of Bayesian methods has not been so widespread; however, their pedigree is as long. One can find references to Bayesian methods in a monograph on parameter estimation from 1974 [15], and dating from the same year is perhaps the first paper on Bayesian methods for structural dynamic SI [16]. To date, the most systematic and extensive development of Bayesian SI is the result of the work of James Beck and his various collaborators. Beck’s early work on statistical system identification is summarized in [17] and his transition to a Bayesian framework is given in [18]. This paper uses a Laplace approximation to remove the need to evaluate intractable high-dimensional integrals. Later, Beck & Au [19] introduce a *Markov chain Monte Carlo* (MCMC) method as a more general means of computing response quantities of interest represented by high-dimensional integrals. Bayesian methods of model selection are discussed in [20], and the paper also discusses the possibility of marginalizing over different model *classes*. A recent contribution [21] discusses identification and model selection for a type of hysteretic system model—the Masing model. Staying with hysteresis models, the paper [22] considers how MCMC can be used for Bayesian estimation of Bouc–Wen models and discusses a simple model selection statistic. Two recent developments which are of interest are the introduction of probability logic for Bayesian SI [23] and a method for potentially reducing computational expense for MCMC by selecting the most informative training data [24]. Bayesian methods for the system identification of differential equations have also been the subject of recent interest in the context of *systems biology* [25,26] and show considerable promise in the context of structural dynamics.

At this point, it is appropriate to define some notation. Here 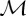 is used to represent a model structure. 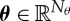 is then used to represent the vector of parameters within that model which requires estimation. Finally, 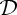 is used to denote a set of observations which one has made about the system of interest, i.e. the measured data. As an example, one may consider the case study which is shown in §5, where one is attempting to create a white-box model of a dynamical system whose response is thought to be greatly influenced by friction effects. In this case, 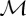 represents the hypothesized equation of motion of the system. Here ***θ*** represents the parameters within the equation of motion which require estimation—in the current example, this includes terms which modulate the level of viscous damping and friction in the system. The data, 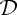, consist of a time history of acceleration measurements which have been taken during a dynamic test. The basic idea of the Bayesian approach to identification is that, by repeatedly applying Bayes’ theorem, one can assess the probability of a set of parameters ***θ*** as well as a model structure 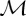 conditional on the data 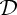 using
1.1
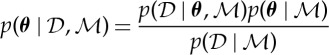
and
1.2
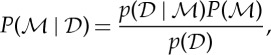
respectively, where
1.3
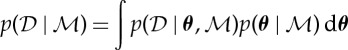
is a normalizing constant which ensures that 
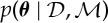
 integrates to unity. This is referred to here as the ‘marginal likelihood’ but can also be described as the ‘model evidence’ (because, as is shown in §2, it can provide evidence for candidate model structures). With equation (1.1), one converts an *a priori* probability density for the parameters ***θ*** into a posterior density having seen the data 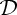. If one desires a point estimate of the parameters, the usual course of action is to choose that which maximizes the posterior probability 
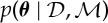
. Now, as the data 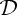 is a constant of the identification problem, one is reduced to maximizing 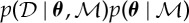. It is often the case at this point, that an uninformative constant (and hence improper) prior 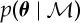 is chosen, and this reduces the problem to that of maximizing 
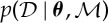
, which is simply the likelihood of the data. The *maximum a posteriori* (MAP) estimate thus becomes maximum likelihood. If one were to further assume that the distribution of any measurement noise was Gaussian (with some extra conditions), the problem essentially becomes one of minimizing a least-squares cost/error function. The sequence of assumptions and approximations discussed above clearly loses much of the benefit of adopting a Bayesian approach in the first place. This paper will emphasize the benefits of a ‘full’ Bayesian methodology.

The layout of the paper is as follows. In §2, the fundamental principles behind a Bayesian approach to system identification are described and the benefits of using MCMC algorithms within a Bayesian framework are emphasized. Sections 3 and 4 are devoted to the description of various MCMC algorithms which can be used to address the issues of parameter estimation and model selection, respectively. These sections are not intended to be a thorough review but, instead, focus on those algorithms which have proved to be particularly useful and/or are based on unique concepts and methodologies.^1^ Finally, in §5, a case study is used to demonstrate how MCMC can be used within a Bayesian framework to generate robust models of nonlinear dynamical systems.

## 2. Bayesian system identification

The problem of SI is easily stated: given measured data from a structure or system, how does one infer the equations of motion which ‘generated’ the data. This problem is not at all easy to solve; it is essentially an inverse problem of the second kind and can be extremely ill-posed even if the underlying equations are assumed to be linear in the parameters of interest [3]. Furthermore, the ‘solution’ may not even be unique. If the equations of motion are not linear in the parameters of interest, the difficulties multiply. Another issue is concerned with *confidence* in derived parameter estimates. This issue is a result of the fact that measurements or data from a system will, in reality, almost always be contaminated by random noise. Given a set of data 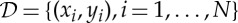 of sampled system inputs *x*_*i*_ and outputs *y*_*i*_, if there is no measurement noise, an identification algorithm should yield a deterministic estimate of the system parameters ***θ***,
2.1
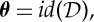
where the function *id* represents the application of the identification algorithm to the data 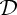. Now, if noise *ϵ*(*t*) is present on the input or output data (or both), ***θ*** will become a random vector conditioned on the data. In this context, one no longer wishes to find an *estimate* of ***θ***, but rather to specify one’s belief in its value. If it is assumed that the noise is Gaussian with (unknown) standard deviation *σ*_*ϵ*_, then the parameter *σ*_*ϵ*_ can be subsumed into ***θ***, and inferred along with the model parameters. In probabilistic terms, instead of equation (2.1) one now has
2.2
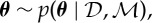
where 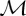 represents the choice of model.

The usual objective of system identification is to provide a predictive model, i.e. one which can estimate or predict system outputs if a different system input is provided. In the probabilistic context described above, the best that one could do is to determine a predictive distribution. Suppose a new input sequence x* were applied to the system, one would wish to determine the density for the predicted outputs
2.3
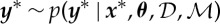
noting all the dependencies.^2^ The mean of this distribution would give the ‘best’ estimates for the predictions and the covariance would allow one to establish confidence intervals for them. However, one notes the presence of the parameter vector ***θ***. In practice, one might use the ***θ*** value corresponding to the mean or the mode of the posterior parameter distribution; however, a truly Bayesian viewpoint on the prediction would require one to marginalize over the parameter estimates, i.e. to derive
2.4
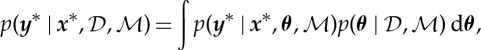
where 
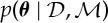
—the posterior parameter distribution—is given by equation (1.1).

This is a very powerful idea: allowing for a fixed model structure, *one is making predictions using an entire set of parameters consistent with the training data*, with each point in the space of parameters weighted according to its probability given the data. In practice, there are considerable problems in implementing the full Bayesian approach, i.e. performing the intractable integral (2.4). One of the main advantages of using MCMC algorithms is that they allow one to *generate samples* from the posterior parameter distribution, even when the geometry of 
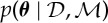
 is complex and its probability density is very concentrated relative to the prior. These samples can then be used as part of Monte Carlo simulations, allowing one to propagate one’s parameter uncertainties without evaluating equation (2.4).

Another potential advantage of a Bayesian approach is that it may be possible to assess the relative evidence for a number of competing model structures. Suppose one believes that the true model structure is one of a finite number 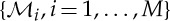 (the discussion here will closely follow [25]). In principle, one could imagine computing the probability of observing the data 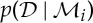, given the particular model structure. If this quantity were available then, by defining a prior probability 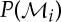 on each model structure, one could use equation (1.2) to select the model with the highest probability. Even more in the spirit of Bayesian inference, one could marginalize over *all possible* model structures weighted according to their probability; in terms of prediction, one would have
2.5
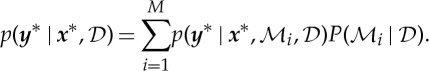
Furthermore, if one appeals to Bayes theorem in the form of equation (1.2) and assumes equal priors on the models, one arrives at a comparison ratio or *Bayes factor*
2.6
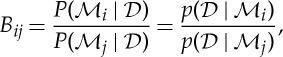
which weights the evidence for two models in terms of marginal likelihoods of the data given the models.

**Figure 1.**
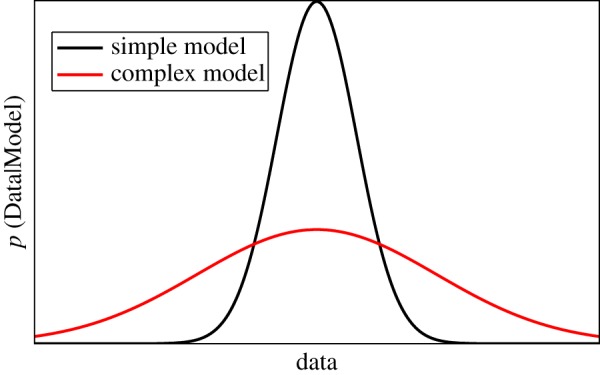
The embodiment of Ockham’s Razor in the marginal likelihood (original explanation described in [7]).

The Bayesian approach to model selection is particularly attractive as the marginal likelihood rewards models for being high fidelity while also penalizing them for being overly complex. By automatically embodying Ockham’s Razor with regard to model selection, it follows that the adoption of a Bayesian approach can help to prevent overfitting. An intuitive explanation of this property is provided by MacKay [7], where it is suggested that a complex model will be capable of replicating a larger range of predictions than a simple model with relatively few parameters. As the probability density function 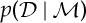 must always be normalized it follows that, in a region where both models are able to replicate the same data, the marginal likelihood will be larger for the simpler model (figure 1). It is in this way that 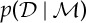 can be used to *provide evidence* for candidate model structures.

For a more detailed explanation, an information theoretic analysis of the marginal likelihood was originally discussed by Beck & Yuen [29] before being generalized in [21]. Noting that 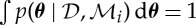, it follows that the logarithm of the marginal likelihood can be written as
2.7
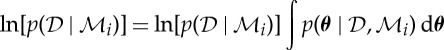

2.8
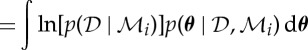

2.9
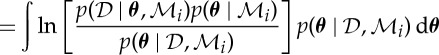
therefore
2.10
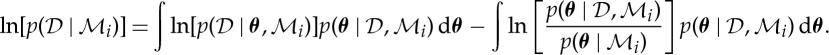
The first term in the above equation is the posterior mean of the log-likelihood which is a measure of the average data fit of model 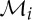. It follows that achieving a good fit to the training data will provide evidence for a candidate model structure. The second term in equation (2.10) represents the relative entropy between the prior and posterior. The marginal likelihood therefore penalizes models which are ‘complex’ where, a complex model is defined as that which is able to extract large amounts of information about the parameters ***θ*** from the data 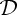. It is important to note that the marginal likelihood is a function of the prior 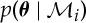
—it is possible to alter the evidence for 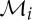 by altering the prior distribution while maintaining the same model structure.

Unfortunately, the marginal likelihoods themselves (equation (1.3)) are often analytically intractable and numerically challenging because of their high-dimensional nature [26]. While one can resort to less informative model selection indicators which are simpler to compute (for example, as used in [22], a Bayesian generalization of the Akaike Information Criterion (AIC) [30] known as the *Deviance Information Criterion* (DIC)), it will be shown in §4 that there now exist MCMC methods which can be used to estimate the marginal likelihoods of different models/generate samples directly from 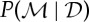.

As a final comment on the issue of model selection, it should be noted that there are already examples of the use of Bayesian strategies for model selection in the structural dynamics literature, the ‘Ockham factor’ defined in [19] being one of these. In [31], the authors use a Bayesian model screening approach in order to determine the appropriate nonlinear terms to include in a system model. The book [32] discusses Bayesian model selection in some detail.

## 3. Markov chain Monte Carlo for the posterior parameter distribution

The first set of algorithms reviewed here are those which are designed to generate samples from the posterior parameter distribution (equation (1.1)), while circumventing the need to evaluate the marginal likelihood (equation (1.3)). These methods involve the creation of an ergodic Markov chain—a Markov chain whose probability distribution tends to a functional form which is independent of time—which evolves through the parameter space (see [33] for a comprehensive discussion on the convergence of Markov chains). Simply stated, MCMC involves ‘forcing’ the stationary distribution of the Markov chain to be equal to (or at least proportional to) some target distribution such that, having allowed the chain to become stationary, it is effectively generating samples from the target. In the context of this section, the target distribution is 
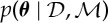
.

Throughout the following text, one’s target distribution is written as *π*(***θ***)=π*(***θ***)/*Z* where π* is used to denote the unnormalized distribution and *Z* is the corresponding normalizing constant.

### (a) The Metropolis algorithm

The Metropolis algorithm was originally proposed in [34], was later generalized by Hastings in [35] and is one of the most established MCMC algorithms. With a Markov chain whose current state is ***θ***^(*i*)^, the first step of the Metropolis algorithm involves probabilistically proposing a new state ***θ***′. This proposal is generated from a probability density function *q*(***θ***′|***θ***^(*i*)^) which is conditional on the current state of the chain and, for the sake of simplicity, will be assumed to be symmetrical and centered on ***θ***^(*i*)^. This proposal then becomes the new state of the Markov chain *with probability*
3.1
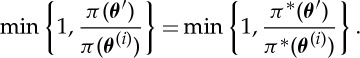
If accepted, the new state of the Markov chain is ***θ***^(*i*+1)^=***θ***′ otherwise ***θ***^(*i*+1)^=***θ***^(*i*)^. The probability of making the transition from some state ***θ*** to the region ***θ***′ d***θ***′ can be written as
3.2
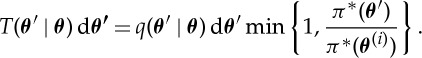
The first point to note is that, by using such a transition, one satisfies the condition known as *detailed balance*:
3.3
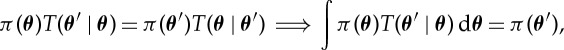
(noting that 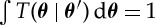) which shows that the stationary distribution of the Markov chain is equal to the target. The second point to note is that, to evaluate the acceptance probability (equation (3.1)), one does not need to know the normalizing constant *Z*. This makes the Metropolis algorithm particularly well suited to Bayesian inference problems as it allows one to sample from 
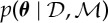
 without having to evaluate the marginal likelihood.

**Figure 2.**
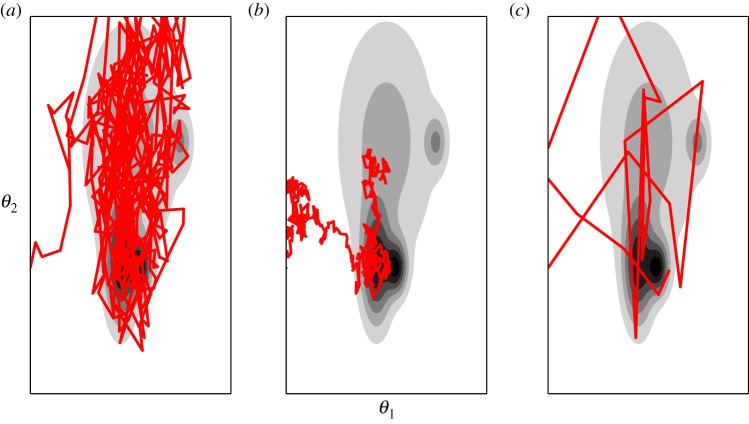
(*a*–*c*) Sampling from a two-dimensional distribution using the Metropolis algorithm.

Figure 2*a* shows an example of the Metropolis algorithm generating samples from a two-dimensional target distribution. It is clear that the Markov chain must go through a transitionary period (known as the ‘burn in’) before it converges to its stationary distribution—the samples generated during this time will need to be discarded. Figure 2*b*,*c* shows what can happen if one selects proposal densities which are have too small or too large variance. With a small proposal density, the Markov chain will take a very long time to converge to its stationary distribution while, with a large proposal density, the majority of the proposed states are rejected and the resulting samples from the Markov chain are highly correlated. The efficiency of the Metropolis algorithm is therefore highly dependent on the tuning of the proposal density *q*. A final point worth noting is that, when using the Metropolis algorithm to generate samples from 
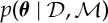
, the use of large data sets may cause numerical issues when one is evaluating the acceptance probability. This can easily be overcome by simply using the logarithm of equation (3.1), such that one then only needs to evaluate the logarithm of the posterior parameter distribution.

### (b) Hybrid Monte Carlo

Hybrid Monte Carlo (also referred to as Hamiltonian Monte Carlo (HMC)) [36] is an MCMC method which is designed to explore parameter spaces more efficiently that the Metropolis algorithm. To facilitate an understanding of HMC, one must envisage that the *i*th element of the parameter vector ***θ*** represents the displacement of a particle in the *i*th direction. One then introduces a vector of momenta 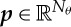 (recalling that *N*_*θ*_ is the number of parameters to be estimated) such that the Hamiltonian of the system is *H*=*K*(***p***)+*V* (***θ***) (where *K* and *V* are the kinetic and potential energies, respectively). Introducing a fictitious ‘time’ variable *τ*, the dynamics of the system can then be evolved through *τ* according to
3.4
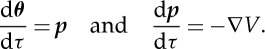
Writing *p*=|***p***|, the key here is to define the kinetic and potential energies as
3.5
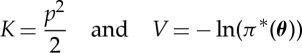
such that
3.6
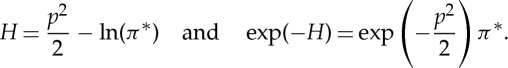
As a result, if one targets the distribution 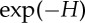 and then simply omits the samples of *p*, one will be left with samples of ***θ*** from the target *π*.

To generate a candidate state {***p***′,***θ***′} from the current state ***θ***^(*i*)^, one must first generate an initial momenta 
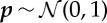
 (noting that this is actually a direct sample from 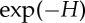). The Hamiltonian of this current state is written as *H*^(*i*)^. The system is then allowed to evolve according to equation (3.4) for a certain amount of ‘time’, until it reaches some state {***p***′,***θ***′} which has Hamiltonian *H*′. As with the Metropolis algorithm, this state is then accepted *with probability*
3.7
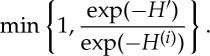


From the above equation, it is clear that, if the dynamics of the system are modelled perfectly, then the new state will always be accepted (as the Hamiltonian must remain constant). In practice, however, the evolution of the system according to equation (3.4) must usually be conducted numerically (usually using finite difference estimates of ∇*V*), and so the Hamiltonian will alter as a result of numerical error. In [36], it is shown that one can still obey detailed balance (and therefore generate samples from 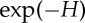) so long as the dynamics of the system are reversible. This can be guaranteed by using the ‘leapfrog’ numerical integration technique (see [27] for more details).

The ability of Hybrid Monte Carlo to ‘generate momentum’ during the proposal process can allow it to conduct efficient explorations of the parameter space relative to the Metropolis algorithm (reference [27] gives a clear explanation of this physical analogy). However, its successful implementation sometimes requires careful tuning of parameters in the leapfrog algorithm, as well as the parameters which dictate how long the system must evolve before a proposal is generated. Furthermore, the need to repeatedly evaluate the posterior distribution to obtain estimates of ∇*V* can make the algorithm computationally expensive. It has however, been successfully applied to various structural dynamics problems in [37–39].

### (c) Simulated annealing

**Figure 3.**
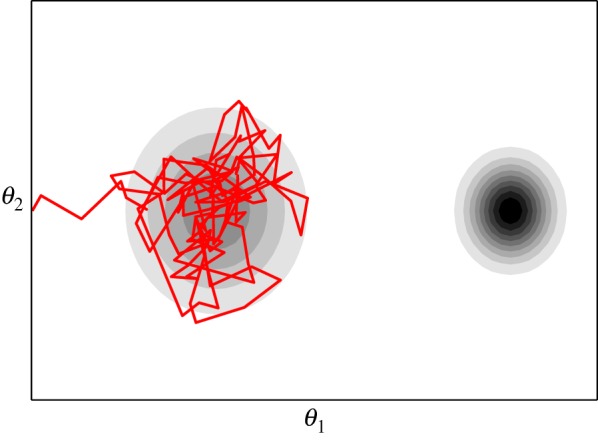
MCMC becoming stuck in a ‘local trap’.

Figure 3 shows a Markov chain which has become ‘stuck’ in a local region of high probability density which has prevented (or at least reduced the probability of) it reaching the globally highest region of probability density. Such local regions are referred to as ‘local traps’. Simulated annealing, which was originally proposed as an optimization algorithm in [40], is a powerful method which not only provides information which can be used to tune the Metropolis algorithm, but which also reduces the probability of local trapping. This is because it initially begins with proposals which allow large steps in the parameter space—the proposal variance is then reduced, thus allowing refinement within a given mode.

Consider the situation where, using the Metropolis algorithm, one aims to generate samples from the target 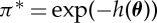. With simulated annealing, one proceeds by targeting the sequence of distributions
3.8
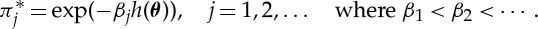
The parameter *β* is referred to as the inverse ‘temperature’ (thus drawing an analogy between *π*_*j*_ and a Boltzmann distribution). One begins by targeting a distribution with a low value of *β* (high temperature) before steadily ‘cooling’ the system until *β*=1, simulating the process of annealing. This essentially means that the ‘fine details’ of the target distribution are introduced gradually and that, at high temperatures, the Markov chain is able to traverse the parameter space relatively freely compared to when *β*=1. It is this which allows the Markov chain to easily escape local traps and converge to the globally optimum region of the parameter space.

When attempting to generate samples from the posterior parameter distribution specifically, a more sophisticated version of simulated annealing involves targeting
3.9
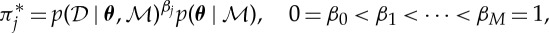
which allows one to facilitate a smooth transition from the prior to the posterior parameter distributions. The strictly increasing sequence of *β* vales (the ‘annealing schedule’) is crucial to the success of the algorithm. Annealing too fast can result in the Markov chain becoming stuck in local traps while annealing to slowly will incur unnecessarily large computational costs. While there are algorithms which feature adaptive annealing schedules (e.g. [27,41–43]), they are not reviewed in this paper.

Simulated annealing has proved to be a very successful methodology and has directly influenced the development of algorithms such as Simulated Tempering [44,45], Exchange Monte Carlo [46], adaptive variants of the Metropolis algorithm [19], Transitional MCMC [42], AIMS [43] and Data Annealing [47] (where the annealing process is instigated through the gradual introduction of data points into the likelihood).

## 4. Markov chain Monte Carlo for the posterior model distribution

The algorithms described in §3 are all designed to generate samples from the posterior parameter distribution while circumventing the need to evaluate the marginal likelihood. While these methods are undoubtedly powerful, they do not allow one to evaluate the posterior model distribution and so can only be used to evaluate what is commonly referred to as the ‘first level of Bayesian inference’. Here, three algorithms are described which, using quite different methods, can be used to address *both* levels of inference—parameter estimation and model selection.

### (a) Transitional Markov chain Monte Carlo

The Transitional MCMC (TMCMC) algorithm was presented in [42]. As with simulated annealing, it involves targeting the sequence of distributions defined by equation (3.9).

Consider the case where one has *N* samples from *π*_*j*_, which are denoted 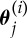, *i*=1,…,*N* (when TMCMC is initiated these would be samples from the prior). One then uses a technique very similar to importance sampling to target the next distribution *π*_*j*+1_. Specifically, one calculates the ‘importance weights’ and ‘normalized importance weights’ of each sample using
4.1
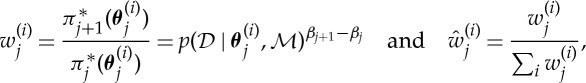
respectively. With standard importance sampling, one would then ‘resample’ by assigning 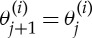 with probability 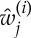. If left to continue in this manner, the algorithm would suffer from the well-known degeneracy problem (a phenomenon often associated with the particle filter), and the set of samples would become dominated by relatively few, highly weighted samples.

To overcome this issue, TMCMC considers each resampled value 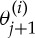 as the starting point of a Markov chain. The Markov chains evolve according to the Metropolis algorithm, each targeting *π*_*j*+1_. The probability that a Markov chain will ‘grow’ is determined by the normalized importance weight of its initial sample. The advantage of this approach is that, by simultaneously growing Markov chains in high probability regions of *π*_*j*+1_, one is able to generate samples from distributions with multiple modes. Once the Markov chains have generated a sufficient number of samples from *π*_*j*+1_, the process is simply repeated until one is left with samples from 
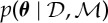
.

With regard to estimating the marginal likelihood, if one denotes ***w***_*j*_ as a vector of importance weights, then from the property that
4.2
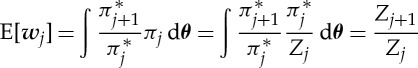
it follows that 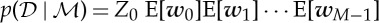. As a result, by estimating the expected value of the importance weights at each stage of the algorithm, one can approximate the marginal likelihood.

As well as being able to sample from distributions with multiple modes and estimate the marginal likelihood, its reliance on the simultaneous growth of multiple Markov chains makes TMCMC suitable for parallel processing [48]. Furthermore, it is also shown in [42] that, by selecting values of *β* which ensure that the coefficient of variation of the importance weights remain within predefined limits, the algorithm is also able to generate an adaptive annealing schedule which prevents large changes in the geometry of the target distribution occurring.

As a result of these benefits, TMCMC has become a popular algorithm which has been applied to many engineering problems (e.g. [49–53]) and has helped to inspire other algorithms such as AIMS [43] (which is not discussed here).

### (b) Reversible jump Markov chain Monte Carlo

Reversible jump MCMC (RJMCMC) [54,55] is unique in that it does not attempt to evaluate 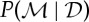 separately for each individual model structure; instead, MCMC is used to target the distribution 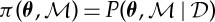. This ‘joint posterior’ is the product of the posterior parameter and model distributions and can be expanded using Bayes’ theorem to gain
4.3
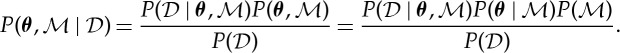
In the following text, ***x*** is used to denote the current state of a Markov chain. In the context of algorithms which generate samples from the posterior parameter distribution only, ***x*** is simply equal to ***θ***, the current position of the Markov chain in the parameter space. For RJMCMC—where the Markov chain is being used to generate samples from the joint posterior (equation (4.3))—the current state of the Markov chain is ***x***={***θ***_*k*_,*k*}, where *k* indexes the model structures and ***θ***_*k*_ represents the current parameter estimates of the *k*th model.

Guaranteeing that the resulting Markov chain will obey detailed balance (and therefore have a stationary distribution equal to the target) is complicated by the fact that different model structures will usually feature different numbers of parameters and, as result, RJMCMC involves the propagation of a Markov chain across parameter spaces of *varying dimension*.

Consider the situation where the algorithm’s current state is 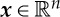 and the state 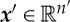 is proposed via
4.4
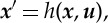
where *h* is a user-defined function and ***u*** is an auxiliary random variable sampled from a distribution *g*(***u***); as will be shown, the variable ***u*** allows the preservation of dimension of the Markov chain and ultimately assures detailed balance.

In [54,55], it is demonstrated that detailed balance will hold if
4.5
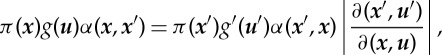
where ***u***′ is a random variable, sampled from *g*′(***u***′), which will facilitate the proposal of ***x*** from ***x***′. Equation (3.3) highlights an issue which RJMCMC must overcome if it is to generate samples from the joint posterior. Specifically, for detailed balance to hold, it must be ensured that the mapping from (***x***,***u***) to (***x***′,***u***′) is diffeomorphic. To address this a ‘dimension matching’ procedure is employed. In the current example, where 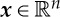 and 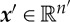, this involves ensuring that 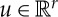 and 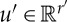 are chosen such that *n*+*r*=*n*′+*r*′.

It is then relatively easy to show that, to ensure that equation (4.5) is satisfied, one must set the acceptance probability, *α*, equal to
4.6
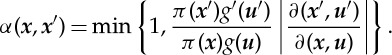
Practically, before RJMCMC can be used one has to outline a set of possible ‘moves’ which collectively can allow the Markov chain to transition from any state ***x*** to any other state ***x***′ (perhaps in more than one step). This usually involves constructing a ‘birth’ and ‘death’ move which, respectively, allow the Markov chain to propose states in models with more or less parameters than the current model structure. Finally, one must also define an ‘update’ move which allows the Markov chain to explore the parameter space of the current model structure (this can be achieved simply by using the Metropolis algorithm). In each iteration of RJMCMC, each of these moves are attempted with a user-defined probability.

The obvious advantage of RJMCMC is that is allows one to investigate the probability of all the competing model structures simultaneously—one does not have to obtain estimates of 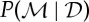 for each model separately. Examples of RJMCMC being applied to mechanical engineering problems can be found in [56–58].

### (c) Nested sampling

Nested sampling [59,60] is unlike the other algorithms discussed here as, rather than generating samples from the posterior parameter distribution, it is specifically designed to estimate the marginal likelihood. It involves noting that the required integral (equation (1.3)) can be viewed as
4.7
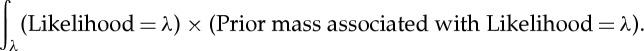
One then defines *X* as the being the prior mass enclosed within the contour where the likelihood is larger than λ:
4.8
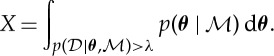
It is then assumed that there exists a function λ=*L*(*X*) which, if one is given *X*, will reveal the corresponding value of λ. When *X*=0 it is clear that there will be no prior mass within the contour defined by 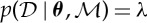 (implying that λ must be larger than 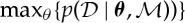. *L*(*X*) is then a decreasing^3^ function of *X* until, when *X*=1, λ must be equal to zero as the entire prior mass is now contained in the contour defined by 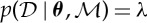.

From equation (4.8), it follows that d*X* represents the prior probability mass 
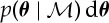
 associated with the contour where the likelihood is equal to λ, ergo
4.9
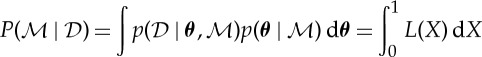
and a difficult multi-dimensional integral has been reduced to a simple one-dimensional integral.

The Nested sampling algorithm begins with *N* samples {***θ***^(1)^,…,***θ***^(*N*)^} from the prior. One then locates the sample ***θ***^(*k*)^ which resulted in the lowest value of the likelihood (denoted 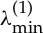). The corresponding value of *X* (written as *X*^(1)^) is estimated according to
4.10
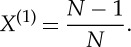
One then replaces ***θ***^(*k*)^ with a sample which has been generated from the prior and is subject to the constraint that the resulting likelihood is larger than 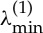 (one may try to achieve this using the Metropolis algorithm or other MCMC methods). This procedure is repeated until a series of *X* values and the corresponding *L*(*X*)=λ have been obtained—standard numerical methods can then be used to estimate 
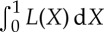
.

While nested sampling is an elegant algorithm, the need to generate samples from the prior subject to constraints on the likelihood can be difficult and, as such, it has not been widely adopted within the context of structural dynamics (although it was used in [61]). It is included here as it provides an interesting method of estimating the marginal likelihood which is fundamentally different from TMCMC or RJMCMC and, with further development, may become more ubiquitous within structural dynamics.

## 5. Case study

The case study shown here was originally conducted as part of a collaborative project with the University of Southampton (full findings are published in [62]); it is included here as it clearly demonstrates how using MCMC methods within a Bayesian framework can be used to quantify and propagate the uncertainties involved in modelling nonlinear dynamical systems.

**Figure 4.**
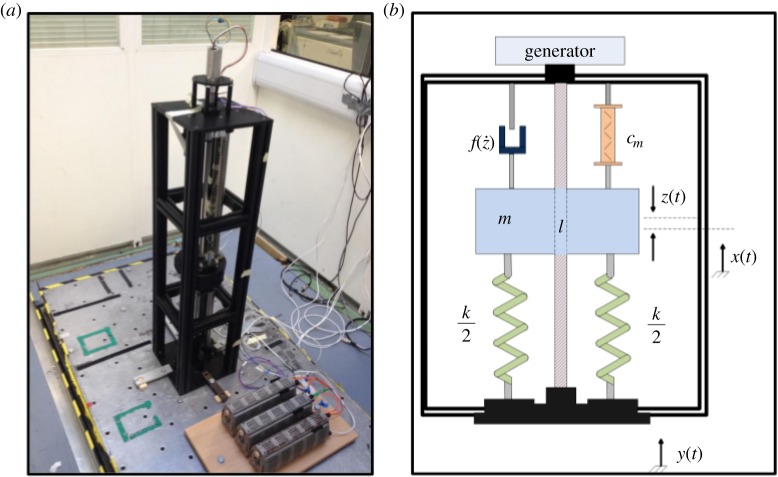
(*a*) Test rig and (*b*) schematic of rotational energy harvester at the University of Southampton Institute of Sound and Vibration.

Figure 4*a* shows a rotational energy harvester—a device which, via a ball–screw mechanism, is designed to convert low-frequency translational motion into high-frequency rotational motion (which can then be transformed into electrical energy). The device is mounted on a electro-hydraulic shaker while accelerometers are attached to the shaker and the oscillating mass (for a more detailed description of the experiment, see [62,63]). With the measured inputs and outputs (*x* and *y*) being the acceleration of the base and mass, respectively, the aim was to use a set of experimentally obtained data to infer a robust model of the device.

Referring to the schematic in figure 4*b*, the equation of motion of the energy harvester is
5.1
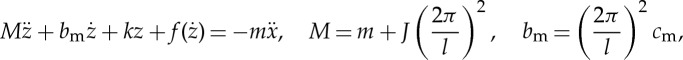
where *z*=*y*−*x* is the relative acceleration between the mass and base, *l* is the ball screw lead, *c*_*m*_ is mechanical damping, *k* is spring stiffness, *m* is the oscillating mass and *J* represents the system’s moment of inertia. The function 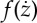 is a friction model which is to be identified. In this case, two models, 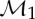 and 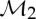, were considered. With the first, it was assumed that all of the parasitic losses in the device could be modelled using a linear viscous damper (which is equivalent to setting 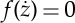) while, with the second, a hyperbolic tangent friction model 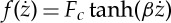 was hypothesized. Assuming that *M*, *k* and *l* were already estimated with sufficient accuracy and employing a Gaussian likelihood with standard deviation *σ*_*ϵ*_, the identification of models 1 and 2 involved estimating the parameter vectors {*c*_m_,*σ*_*ϵ*_} and {*c*_m_,*F*_*c*_,*β*,*σ*_*ϵ*_}, respectively. Aside from obtaining probabilistic estimates for the parameters in each model, the aim here was to establish whether it is worth including the additional complexity of the hyperbolic tangent friction model.

**Figure 5.**
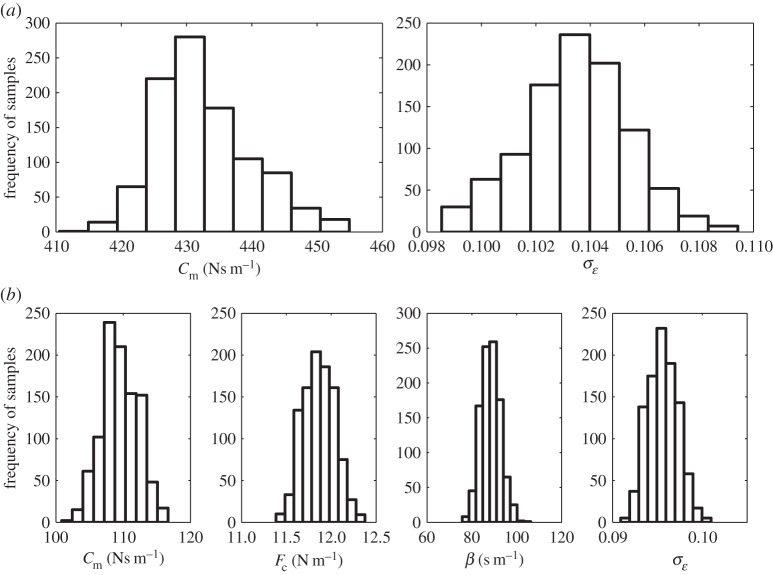
MCMC samples generated for model 1 (*a*) and model 2 (*b*).

**Figure 6.**
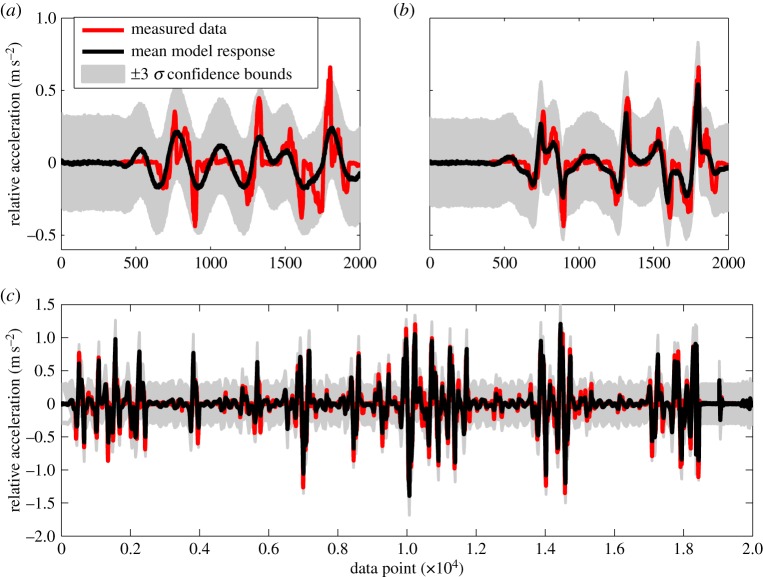
The ability of (*a*) model 1 and (*b*) model 2 to replicate the training data. (*c*) Shows predictions about previously ‘unseen’ data using model 2.

Using TMCMC to generate 1000 samples from the posterior parameter distribution of each model, figure 5 shows the histograms of the resulting samples (where row 1 is model 1 and row 2 is model 2). Using these samples as part of Monte Carlo simulations, figure 6*a*,*b* shows the ability of model 1 and model 2 to replicate the training data (the filled grey regions in figure 6 represent 3*σ* confidence bounds). These results seem to indicate that the additional complexity of model 2 has allowed it to form a more accurate representation of the training data. Using TMCMC to analyse the marginal likelihood, the finding that 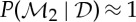 confirms that model 2 is preferable. Finally, figure 6*c* shows the ability of model 2 to replicate a set of ‘unseen’ acceleration time history (data which were not used to train the model).

It is interesting to note that, although model 2 provides a better fit to the data, the confidence bounds on the predictions made by each model are of a similar magnitude. This is essentially due to a poor ‘initial phrasing’ of the problem. Specifically, when the likelihood was defined, it was assumed that the probability of witnessing each data point was a Gaussian distribution, centred on the prediction made by the model and with variance 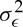. The choice of a Gaussian distribution is justified somewhat by the Principle of Maximum Entropy [64] from which one finds that, having assumed the first 2 moments of the likelihood, the Gaussian distribution is that which minimizes the amount of additional information that must be assumed. However, having completed the analysis using such a likelihood, it can be observed that model 2 actually appears better able to replicate the experiment when at low amplitudes. From this, one may conclude that the probability of witnessing a data point, conditional on the model, actually varies with amplitude. As a continuation to this study one could propose a more complex likelihood before repeating the analysis. Potentially, one could then adopt a Bayesian approach to the selection of different likelihoods, thus preventing the selection of overly complex ‘error-prediction’ models (e.g. [65]).

## 6. Future work

Ultimately, with each sample generated using MCMC requiring a model run, the applicability of MCMC to Bayesian system identification problems is limited by computational cost. This places several restrictions on the types of problems which can be addressed. For situations where one’s model is expensive, a current stream of research is aimed towards the development of MCMC algorithms which are suitable for large-scale parallelization [66], and those which are able to reduce computational cost via the exploitation of interpolation methods (see [67] for example, where kriging is integrated into TMCMC). Further interest has been directed towards the scenario where one is confronted with large datasets from which to infer models. The work [24] proposes a method which allows the selection of small, highly informative subsets of data while, in [47,68], MCMC methods are proposed which allow the tracking of one’s parameter estimates as more data are analysed (helping to establish when a sufficient amount of data has been used).

## 7. Conclusion

In this paper, the authors have presented arguments for the adoption of a Bayesian framework for the system identification of nonlinear dynamical systems in the presence of uncertainty. Specifically, it has been highlighted how a Bayesian approach allows one to realize probabilistic parameter estimates in the presence of measurement noise, select high fidelity models which are not overfitted and make predictions which are marginalized over one’s parameter estimates and, in some cases, over a set of candidate model structures. It is then shown how many of the potential difficulties with such an approach can be overcome through the use of Markov chain Monte Carlo (MCMC) algorithms. A brief tutorial/review of six different MCMC algorithms is then given, each of which has been chosen because it has either proved to be particularly useful and/or is based on unique concepts and methodologies. The paper finishes with a case study, where an MCMC algorithm is demonstrated within a Bayesian framework to realize a model of a nonlinear, rotational energy harvester.

## Supplementary Material

Training data

## Footnotes

1 For a more detailed description of various MCMC algorithms, the technical report by Neal is recommended [27] while, for the interested reader, it is worth noting that [28] is an impressive Python resource for coding MCMC schemes.

2 Without loss of generality, the reader can regard ***x**** and ***y**** as a set of samples, a set of variables or both.

3 Technically, it is assumed to be strictly decreasing such that there is a 1–1 relationship between *X* and λ—see [60] for more details.

## Data accessibility

The training data are available in the electronic supplementary material.
